# Role of Intelligence in Posttraumatic Stress Disorder Development: A Systematic Review

**DOI:** 10.3390/jintelligence14050073

**Published:** 2026-05-01

**Authors:** Marcos Lacombe, Ana M. Pérez-García, Isabel Ramírez-Uclés

**Affiliations:** 1 Escuela Internacional de Doctorado (EIDUNED), Universidad Nacional de Educación a Distancia (UNED), 28040 Madrid, Spain; mlacombe1@alumno.uned.es; 2 Department of Personality, Assessment and Psychological Treatments, Universidad Nacional de Educación a Distancia (UNED), 28040 Madrid, Spain; aperez@psi.uned.es

**Keywords:** posttraumatic stress disorder, intelligence, intelligence quotient, systematic review

## Abstract

Posttraumatic stress disorder (PTSD) has been associated with a range of cognitive alterations; however, the relationship between PTSD and intelligence remains unclear. The aim of this systematic review was to examine potential differences in intelligence associated with exposure to traumatic events and/or a diagnosis of PTSD in adults aged 18 years and older. A systematic search was conducted across three major academic databases—PsycINFO, MEDLINE, and Web of Science—to identify empirical studies assessing intelligence or closely related cognitive constructs in individuals with PTSD or a history of trauma exposure. After applying predefined inclusion criteria, 12 studies were included in the review. Intelligence was assessed using various psychometric instruments, encompassing both global intelligence measures and specific domains such as verbal intelligence and vocabulary. Overall, the findings consistently indicated a negative association between intelligence and PTSD, with lower intelligence scores more frequently observed among individuals with PTSD or significant traumatic exposure. Due to the correlational design of the studies included, causal relationships cannot be established. Consequently, it remains unclear whether intelligence is affected by PTSD or whether higher intelligence may serve as a protective factor against the development or severity of the disorder. From an intelligence research perspective, these findings highlight cognitive ability as a key factor associated with vulnerability and resilience in trauma-related psychopathology.

## 1. Introduction

Intelligence is a concept extensively studied across multiple disciplines, which makes providing a single definition challenging (Neisser et al., 1996). Broadly, intelligence is understood as “a mental activity directed toward purposive adaptation to, and selection and shaping of, real-world environments relevant to one’s life” (Sternberg, 1985, p. 45). This definition is aligned with “the aggregate or global capacity of the individual to act purposefully, to think rationally, and to deal effectively with his environment” (Wechsler, 1958, p. 7).

Beyond this general understanding, several theories have expanded the concept, emphasizing its multifaceted nature. Gardner (1987) proposed the theory of multiple intelligences, which identifies diverse types of intelligence, including linguistic, logical-mathematical, spatial, musical, interpersonal, intrapersonal, naturalistic, and kinesthetic. Individuals may demonstrate varying strengths and weaknesses across these domains. Recent research further highlights that intelligence interacts closely with personality traits and broader psychopathological profiles, influencing both adaptive and maladaptive outcomes (Colom & Ma, 2024). In this context, understanding intelligence requires not only measuring cognitive capacity but also considering its interactions with emotional, social, and personality factors.

At a psychometric level, it is also important to distinguish between general intelligence (g), typically operationalized through global IQ scores derived from standardized instruments such as the WAIS, and specific cognitive abilities (executive functions, working memory, attention, processing speed, or verbal abilities) (Spearman, 1904). Although these domains are interrelated, they represent partially distinct constructs. This distinction is particularly relevant in the context of PTSD research, where some studies assess full-scale IQ while others focus on specific neurocognitive domains. Throughout this review, the term “intelligence” is used in accordance with the operationalization adopted in each included study, and this heterogeneity is considered in the interpretation of findings.

Intelligence is commonly operationalized through the Intelligence Quotient (IQ), validated across diverse populations through psychometric instruments designed to assess general intellectual functioning. Among these, the Wechsler Adult Intelligence Scale (WAIS) conceptualizes IQ as a hierarchical and multifactorial construct encompassing verbal comprehension, perceptual reasoning, working memory, and processing speed, yielding a Full Scale IQ score (Wechsler, 2008). As a multidimensional capacity, intelligence is shaped by skills, environmental influences, and life experiences, and it affects both cognitive and psychosocial functioning.

Importantly, IQ scores obtained from different instruments are not directly interchangeable, as they rely on distinct theoretical frameworks and cognitive domains. Consequently, IQ measured with the WAIS differs conceptually and psychometrically from IQ derived from other instruments, such as the Stanford–Binet Intelligence Scales or Raven’s Progressive Matrices (Deary, 2012; Neisser et al., 1996).

Moreover, intelligence contributes to how individuals perceive, interpret, and respond to potentially traumatic experiences. For example, maltreatment-related trauma has been shown to impair academic achievement through its effects on cognitive functioning, demonstrating the interplay between cognitive capacity and environmental stressors (Ogata, 2017).

Similarly, emotional intelligence and cognitive–emotional processing may shape how older adults respond to social and attachment-related challenges, further illustrating the complex ways in which cognitive capacities interact with life experiences (Durao et al., 2023).

From this perspective, intelligence is not merely an outcome variable, but a core cognitive resource that shapes vulnerability, adaptation, and recovery following exposure to traumatic events.

Potentially traumatic events are defined by the American Psychiatric Association (2013) as exposures involving actual or threatened death, serious injury, or threats to physical integrity. Such exposures can be direct, witnessed, or learned about through a close connection. In addition to widely recognized traumatic events such as combat exposure or sexual violence, research has increasingly examined less frequently discussed trauma contexts. For example, the experience of committing homicide, although rarely addressed in mainstream trauma research, has been associated with substantial levels of posttraumatic stress symptoms. A recent meta-analysis found that a considerable proportion of individuals who had committed homicide met criteria for full or partial PTSD, highlighting that traumatic stress reactions are not limited to traditional victim-based scenarios (Badenes-Ribera et al., 2021). These findings suggest that trauma exposure may encompass a broader range of experiences than is often assumed, reinforcing the need for nuanced conceptualizations of potentially traumatic events in adult populations.

The impact of these events depends on how individuals process them, with approximately 15% of exposed individuals developing trauma-related symptoms (Bonanno & Mancini, 2012; Tedeschi & Calhoun, 2004).

Sociodemographic variables such as sex, age, educational attainment, and socioeconomic status have consistently been associated with differential risk for PTSD (Tolin & Foa, 2006). For example, women tend to show higher lifetime prevalence rates of PTSD despite lower exposure to certain types of trauma, and lower educational attainment has been linked to increased vulnerability (Brewin et al., 2000). These factors may be theoretically connected to intelligence, insofar as educational opportunities, cognitive reserve, and social resources can influence both measured intelligence scores and coping capacities following trauma. Therefore, understanding the relationship between intelligence and PTSD requires considering these overlapping sociodemographic determinants.

Posttraumatic Stress Disorder (PTSD) is the mental health disorder most closely associated with potentially traumatic events. PTSD manifests as a persistent and overwhelming emotional response to trauma, encompassing experiences such as natural disasters, war, or physical and sexual abuse, and significantly affects cognitive, emotional, and behavioral functioning (American Psychiatric Association, 2013).

The interplay between intelligence and PTSD has been a central topic in trauma research and clinical psychology. Individuals with higher cognitive abilities may better analyze, reflect on, and structure their understanding of traumatic experiences, facilitating adaptive coping strategies such as information-seeking, social support, and engagement in activities that promote mental health and well-being (Brewin et al., 2000; Charney, 2004; Ozer et al., 2003).

Large-scale evidence supports this view; one UK Biobank study found that individuals with high general intelligence were significantly less likely to exhibit PTSD than those with average intelligence (Williams et al., 2023). Similarly, pre-trauma cognitive ability strongly predicted PTSD risk in a twin study: individuals in the highest ability quartile had approximately 48% lower PTSD risk than those in the lowest quartile (Kremen et al., 2007).

Emotional intelligence can further modulate coping and attachment-related processes, highlighting the broader influence of cognitive–emotional capacities on trauma outcomes across the lifespan (Durao et al., 2023). Conversely, intelligence may also confer vulnerability: higher cognitive capacities can be associated with increased sensitivity to traumatic memories and heightened risk for PTSD symptoms. Additionally, exposure to trauma can impair cognition, demonstrating the bidirectional relationship between PTSD and intellectual functioning (Yehuda, 2002). Importantly, although some studies suggest that lower premorbid intelligence may function as a vulnerability factor, the correlational nature of most available research precludes firm causal inferences (McEwen, 2007). The relationship may also be influenced by third variables such as depressive symptoms, chronic stress, comorbid psychiatric conditions, or educational background (Rock et al., 2014). Therefore, caution is warranted when interpreting statements implying that intelligence directly protects against or is reduced by PTSD.

Individual differences, including personality traits and perceptions of intelligence, may modulate these dynamics, suggesting that intelligence can both buffer and exacerbate vulnerability to trauma-related disorders depending on context (Colom & Ma, 2024; Silverio et al., 2023).

While research has extensively explored the relationship between PTSD and cognitive functioning in child and adolescent populations, adult populations remain comparatively underrepresented. Studies such as Hahnefeld et al. (2022) and Gervasio et al. (2022) have examined non-verbal cognitive development, learning, and trauma symptoms in young children and psychiatric inpatients, demonstrating the importance of developmental perspectives in understanding the cognitive impact of trauma. At the same time, integrating information from pathophysiology, treatment, and biomarker studies underscores the complexity of PTSD and the relevance of cognitive functioning in predicting and managing the disorder (Al Jowf et al., 2025).

In summary, the relationship between intelligence and PTSD is complex, multifaceted, and bidirectional. Intelligence influences how individuals process, cope with, and recover from trauma, while trauma exposure and PTSD can, in turn, affect cognitive and intellectual functioning. A comprehensive understanding of this relationship requires integrating cognitive, emotional, and biological perspectives. The present systematic review aims to synthesize existing literature, address gaps, and elucidate the interactions between intelligence and traumatic experiences, with the goal of informing research, clinical practice, and interventions.

## 2. Materials and Methods

### 2.1. Protocol and Reporting Guidelines

This systematic review was conducted in accordance with the Preferred Reporting Items for Systematic Reviews and Meta-Analyses (PRISMA) 2020 guidelines (Page et al., 2021). The review protocol was registered in the International Prospective Register of Systematic Reviews (PROSPERO; registration number CRD420251156056). The PRISMA checklist is provided in the Supplementary Materials.

### 2.2. Research Question and Framework

The following sections—search structure, research questions, search, and inclusion and exclusion criteria—are all parts of the methodology. For this systematic review, the PECO framework was followed, which includes the following components: identification of the population (P), exposure variable (E), comparative group (C), and observation of outcomes (O). The framework was applied as follows:

P: Adult population.
E: Diagnosis or presence of posttraumatic stress disorder (PTSD) or clinically significant PTSD symptoms.
C: Adults without PTSD or with lower levels of PTSD symptomatology.
O: Measured differences in intelligence, including global IQ and/or specific cognitive domains.

Using this structure, the research question of this review is: Are there differences in intelligence between adults with PTSD and those without PTSD or with lower symptom severity? The objective of this review was to synthesize and critically examine the available scientific literature on the relationship between intelligence and exposure to traumatic events.

### 2.3. Search Strategy

Once the research question was formulated and the protocol registered, a systematic literature search was conducted in the following electronic databases: PsycINFO, MEDLINE, and Web of Science. The search strategy combined terms related to posttraumatic stress disorder and intelligence, using Boolean operators as follows: (“posttraumatic stress disorder” OR “post-traumatic stress disorder” OR “PTSD”) AND (“intelligence” OR “IQ”).

Although the search terms focused specifically on “intelligence” and “IQ,” several included studies operationalized intelligence using standardized psychometric instruments that assess both global intellectual functioning and specific cognitive domains such as verbal comprehension, working memory or processing speed. Therefore, variations in operational definitions were considered during data extraction and synthesis.

### 2.4. Eligibility Criteria

The inclusion criteria required that studies involve adult participants aged 18 years or older. No restrictions were applied regarding the language of publication, and studies from all publication years available in each of the consulted databases were considered eligible.

Studies were excluded if they focused on children or adolescents under the age of 18 or if they did not constitute primary research. Specifically, systematic reviews, meta-analyses, book chapters, books, and doctoral dissertations were excluded from the review.

### 2.5. Study Selection Process

The search identified 2 additional references through other sources. Database searches yielded 381 records from MEDLINE, 907 from PsycINFO, and 1171 from Web of Science. After removing 1162 duplicates, a total of 1297 records were screened. During the screening phase, studies were excluded for the following reasons: use of child or adolescent samples (87 records), focus on other disorders unrelated to PTSD (491 records), scale validation studies (235 records), treatment-efficacy research (418 records), and neurobiological investigations without relevant psychological or cognitive outcomes (29 records).

In total, 37 full-text publications were assessed for eligibility, and 12 studies met the criteria for inclusion in the final systematic review. The remaining 25 full-text articles were excluded primarily due to: absence of intelligence measures (8), lack of a clearly defined PTSD diagnosis or symptom assessment (3), failure to provide a comparative framework allowing evaluation of differences in intellectual functioning (2) or sample age (12).

### 2.6. Data Extraction and Data Items

For each included study, the following sample characteristics included mean age, sex distribution, and whether the population consisted primarily of military or civilian participants. Information regarding PTSD diagnostic criteria or symptom assessment instruments was also recorded. Additionally, details regarding the specific operationalization of intelligence (full-scale IQ scores versus domain-specific subtests) were extracted to allow a more nuanced interpretation of findings. This information is presented in Table 1.

### 2.7. Risk of Bias Assessment

To analyze bias risk, the index proposed by the Cochrane Collaboration (Higgins & Green, 2011) was applied to all included studies. Bias domains assessed included: selection, performance, detection, attrition, reporting, and authors’ acknowledgement of limitations. A detailed summary is provided in Table 2.

Given that most included studies were observational in design, the assessment focused particularly on selection bias, measurement validity of PTSD and intelligence constructs, and completeness of outcome reporting. Risk-of-bias evaluations were conducted independently by two reviewers, and discrepancies were resolved through discussion until consensus was reached. The overall methodological quality of the included studies was considered during interpretation of the findings and is discussed in relation to the strength of the evidence.

### 2.8. Review Protocol

**Figure 1:**
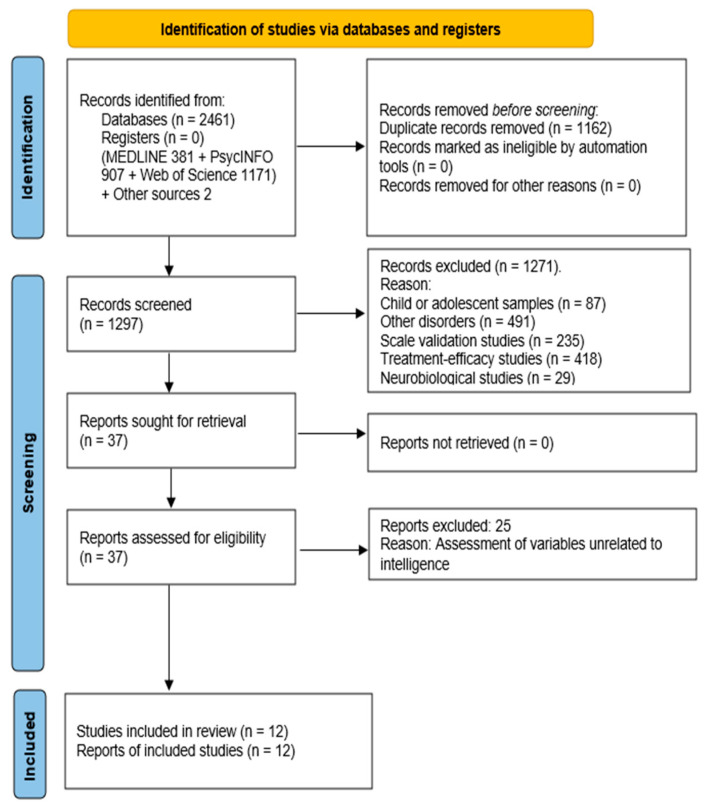
Flow chart depicting the process of selection of papers for final analysis.

Figure 1 presents the PRISMA 2020 flow diagram summarizing the study identification, screening, eligibility, and inclusion process.

## 3. Results

The characteristics of the studies are summarised in Table 1. Out of a total of 12 analysed studies, a sample of up to 1575 participants was extracted, of which 33.4% (526) were diagnosed with PTSD, and 66.6% (1049) were included in the control group. Of the sample, 85.3% (1379) were men, and only 14.7% were women (196).

When analysing the articles individually, McNally and Robinaugh (2011) found the highest representation of women at 100%. In contrast, most studies conducted on combat veterans (Gilbertson et al., 2006; Macklin et al., 1998; McNally & Shin, 1995; Sørensen et al., 2016; Vasterling et al., 2002; Zalmenson et al., 2024) included only men in the samples.

Regarding the countries where the research was conducted, most studies (8) were conducted in the United States (Burriss et al., 2008; Gilbertson et al., 2006; Macklin et al., 1998; McNally & Robinaugh, 2011; McNally & Shin, 1995; Vasterling et al., 1997; Vasterling et al., 2002), with the remainder in Canada (Desrochers et al., 2021), The Netherlands (Olff et al., 2014), Sweden (Emdad et al., 2005), Denmark (Sørensen et al., 2016) and Israel (Zalmenson et al., 2024).

More than two thirds of the studies (8) were conducted with former combat military personnel either in Vietnam (Burriss et al., 2008; Gilbertson et al., 2006; Macklin et al., 1998; McNally & Shin, 1995; Sørensen et al., 2016; Vasterling et al., 2002), the Persian Gulf (Vasterling et al., 1997) or the Middle East (Zalmenson et al., 2024). Police (Desrochers et al., 2021), the general population (Olff et al., 2014), Iraqi refugees (Emdad et al., 2005), and women victims of sexual assault (McNally & Robinaugh, 2011) were also considered. This distribution indicates a marked predominance of military male samples within the available literature, whereas civilian and female populations were comparatively underrepresented.

Various scales were used to measure intelligence. The Wechsler Adult Intelligence Scale (WAIS) and its subtests have been widely used in many studies (Burriss et al., 2008; Desrochers et al., 2021; Gilbertson et al., 2006; McNally & Shin, 1995; Vasterling et al., 1997, 2002). Other scales included the Block Design Test (BDT), Benton Visual Retention Test (BVRT), Raven’s Standard Progressive Matrices (RSPM), Thurstone’s Picture Memory Test (TPMT) (Emdad et al., 2005), Shipley Institute of Living Scale (SILS) (McNally & Robinaugh, 2011), and Cambridge Neuropsychological Test Automated Battery (CANTAB) (Olff et al., 2014). One study utilised different scales, such as the SILS, for its participants (Macklin et al., 1998).

Although several studies employed full-scale IQ measures, others relied primarily on subtests or domain-specific neuropsychological instruments assessing memory, executive functioning, or processing speed. Olff et al. (2014) used CANTAB and made estimations based on other functions, such as memory. Sørensen et al. (2016) and Zalmenson et al. (2024) used a self-designed questionnaire (BPP and IDF IQ assessment). Therefore, the operationalization of “intelligence” varied across studies, with some investigations focusing on global intellectual functioning and others on specific cognitive domains.

Regarding the conclusions drawn by the authors, all studies reported statistically significant differences between the PTSD and non-PTSD populations in terms of intelligence or cognitive performance. Several studies interpreted higher premorbid intelligence as potentially protective and lower intelligence as associated with increased PTSD vulnerability (Gilbertson et al., 2006; Macklin et al., 1998; Sørensen et al., 2016; Zalmenson et al., 2024). However, these interpretations were based on observational and correlational data. Vasterling et al. (2002) reported reductions in measured intellectual performance between pre- and post-combat assessments among individuals who developed PTSD, although the design does not allow firm conclusions regarding causality or directionality. Other studies identified differences in verbal areas (Vasterling et al., 1997) and in both verbal and vocabulary (Burriss et al., 2008) rather than uniform reductions across all cognitive domains.

Finally, significant statistical insignificance was noted in several groups (*n*2 and *n*3) in McNally and Robinaugh’s (2011) study.

### Bias Risk Assessment

To analyse bias risk, the index proposed by the Cochrane Collaboration (Higgins & Green, 2011) was used for the trials conducted by Burriss et al. (2008), Desrochers et al. (2021), Emdad et al. (2005), Gilbertson et al. (2006), Macklin et al. (1998), McNally and Robinaugh (2011), McNally and Shin (1995), Olff et al. (2014), Sørensen et al. (2016) Vasterling et al. (1997), Vasterling et al. (2002) and Zalmenson et al. (2024).

Table 2 provides a summary of the risk of bias assessment for each study, categorizing different types of bias (selection, performance, detection, attrition, reporting, and other biases). Each article has been evaluated based on specific criteria, such as random sequence generation, allocation concealment, blinding, handling of incomplete data, and selective reporting. The risk level is classified as low, high, or not specified, allowing for a comparative analysis of methodological quality across studies.

Selection bias was assessed in two domains: randomised sequence generation (where methods varied among studies), and allocation concealment, which was not specified in any study.

Performance bias related to participant and personnel blinding was only specified by Gilbertson et al. (2006); other studies did not provide such information.

Detection bias concerning blinding of outcome assessors was mentioned only by Emdad et al. (2005); other studies did not specify blinding procedures.

Attrition bias, based on handling incomplete outcome data, was addressed comprehensively in all studies without any indication of bias due to missing data.

Reporting bias, which indicates selective reporting, was indicated by most of the authors, including outcome variables and associations analysed. McNally and Shin (1995) did not report any non-significant elements posing a higher risk of this bias.

Lastly, the acknowledgement of study limitations varied among authors, with Emdad et al. (2005) failing to include such information, elevating their risk in this regard.

Overall, the methodological quality of the included studies can be considered moderate to low, primarily due to limited reporting of allocation procedures, lack of blinding, and insufficient specification of methodological safeguards in several investigations. These limitations should be taken into account when interpreting the reported associations between PTSD and intelligence, as the potential influence of selection bias, measurement variability, and unreported confounding variables cannot be fully excluded.

## 4. Discussion

This systematic review examined the relationship between intelligence and posttraumatic stress disorder (PTSD) in adults, addressing the diversity of perspectives and findings in the scientific literature. Through an exhaustive search across multiple databases, several studies exploring this connection were identified, highlighting the complexity and multifactorial nature of the association between cognitive functioning and trauma-related outcomes. The diversity of methodologies, populations, and intelligence assessment tools underscores the challenges in synthesizing the findings, but also reflects the richness of research approaches in this field. Importantly, the term “intelligence” was operationalized heterogeneously across studies, ranging from full-scale IQ measures to domain-specific neuropsychological tasks assessing memory, executive functioning, or processing speed. Consequently, some findings may reflect differences in specific cognitive domains rather than global intellectual functioning, which should be considered when interpreting the results.

The research highlighted the potential role of intelligence in PTSD, yet it remains necessary to clarify whether intelligence acts primarily as a protective factor, facilitates better recovery, or is itself influenced by exposure to traumatic experiences. The findings indicate a negative and significant association between these two constructs; however, the exact nature of the relationship remains difficult to determine due to the heterogeneity of the included studies. Differences in sample characteristics, assessment tools, trauma types, and timing of evaluations all contribute to variability in the results, emphasizing the need for cautious interpretation.

A notable discrepancy was observed regarding the directionality of the association. Some studies suggested that lower intelligence may predict a higher risk of developing PTSD, whereas higher intelligence may confer a protective effect against trauma-related symptoms (Gilbertson et al., 2006; Macklin et al., 1998; Sørensen et al., 2016; Zalmenson et al., 2024). However, these interpretations are derived primarily from observational and correlational designs, which do not allow definitive causal conclusions. It remains possible that premorbid cognitive ability, educational attainment, socioeconomic status, or co-occurring psychiatric conditions contribute to the observed associations.

Conversely, other research indicated that PTSD itself may contribute to a decline in measured intelligence (Vasterling et al., 2002). Nevertheless, reductions in post-trauma cognitive performance may reflect temporary stress-related impairments, depressive symptomatology, chronic hyperarousal, or methodological differences in assessment timing rather than irreversible changes in general intellectual capacity. These findings highlight the complex bidirectional relationship between cognitive functioning and trauma, suggesting that intelligence may both influence and be influenced by PTSD symptomatology. This complexity is further reflected in the variations observed across specific cognitive domains, including verbal and non-verbal intelligence, problem-solving abilities, and memory functions.

When examining the studies conducted in military and ex-military populations, a relatively consistent pattern may indicate that lower premorbid intelligence may function as a vulnerability factor for the development and severity of PTSD symptoms. Cohortstudies in combat-exposed samples reported that lower baseline cognitive ability was associated with an increased likelihood of later PTSD diagnosis or greater symptom severity. At the same time, some investigations within military contexts also documented reductions in cognitive performance following trauma exposure, particularly in domains such as verbal memory, executive functioning, and processing speed.

However, given the structured training environments, selection processes, and shared occupational stressors characteristic of military populations, these findings may reflect context-specific factors that interact with cognitive functioning. Overall, within military samples, the vulnerability hypothesis appears somewhat more consistently supported, although evidence for post-trauma cognitive changes is also present.

In contrast, findings from civilian populations appear more heterogeneous and less conclusive. Civilian studies showed greater variability in the strength and direction of associations between intelligence and PTSD. Some studies did not find statistically significant relationships in certain cognitive domains, while others suggested that PTSD symptom severity was associated with lower cognitive performance.

The smaller number of civilian studies, together with differences in trauma type, may partly account for this inconsistency. These variations may indicate that the intelligence–PTSD relationship might be moderated by contextual and occupational factors, rather than uniform effects across trauma-exposed groups.

Additionally, several studies indicated that intelligence modulates the severity of PTSD in affected individuals (Emdad et al., 2005; Macklin et al., 1998; McNally & Shin, 1995; Sørensen et al., 2016; Zalmenson et al., 2024). These studies generally agreed that greater severity of PTSD symptoms was associated with lower intelligence scores, or that lower intelligence scores were linked to more severe symptoms. This suggests that cognitive capacity may play a crucial role in coping, adaptive strategies, and recovery, influencing both the presentation and progression of PTSD. Furthermore, the interaction between intelligence and PTSD severity may reflect underlying cognitive resilience mechanisms, such as the ability to organize and integrate traumatic experiences, maintain attention and working memory under stress, and engage in effective problem-solving when confronted with challenging circumstances.

Discrepancies were also noted in specific aspects of verbal intelligence, as reported by Vasterling et al. (1997) and Burriss et al. (2008). Some studies failed to find statistically significant associations between intelligence and PTSD in certain populations or subscales, as in McNally and Robinaugh (2011). These inconsistencies could be attributed to differences in assessment methods, sample sizes, demographic characteristics, or co-occurring conditions. Additionally, several authors (Burriss et al., 2008; Olff et al., 2014) suggested that mood disorders, depressive symptoms, or other affective conditions may mediate or confound the relationship between intelligence and PTSD. This highlights the importance of interpreting intelligence scores within the broader clinical and psychological context, recognizing that trauma does not occur in isolation but interacts with multiple cognitive and emotional processes.

Taken together, the studies reviewed suggest that the association between intelligence and PTSD is influenced by multiple factors, including trauma severity, individual cognitive capacity, symptom presentation, and comorbid conditions. Higher intelligence may be associated with greater cognitive flexibility, problem-solving skills, and coping strategies that could mitigate the psychological impact of trauma. However, such interpretations should be considered tentative, as they may also reflect broader educational, social, and psychological resources correlated with measured intelligence.

Conversely, exposure to severe or prolonged trauma may be associated with a negative effect on cognitive performance, resulting in observable reductions in measured intelligence. The bidirectional and dynamic nature of this relationship underscores the need for nuanced interpretations and for studies that consider multiple dimensions of cognition, including verbal and non-verbal reasoning, executive function, and memory performance.

In addition, the literature reviewed reflects substantial heterogeneity in populations, including military, ex-military, police personnel, and civilian samples. Although a marked predominance of military and male samples was observed. More than two-thirds of the included studies were conducted with combat-exposed military personnel, and over 85% of the total sample consisted of men. This imbalance substantially limits the generalizability of the findings to the broader adult population, particularly to civilian and female populations, whose trauma exposure patterns and psychosocial contexts may differ considerably.

Differences in occupational exposure, training, and stress resilience may account for some of the observed variability, indicating that context-specific factors may influence the intelligence-PTSD relationship. This further complicates attempts to generalize findings and suggests that future studies should explore population-specific moderators, including occupational and environmental influences.

Furthermore, the overall methodological quality of the included studies was moderate to low, with several investigations lacking detailed reporting of allocation procedures, blinding strategies, or comprehensive control of confounding variables. These methodological limitations increase the risk of selection and measurement biases and warrant caution when interpreting the consistency and strength of the reported associations.

Overall, this systematic review highlights the complexity of the relationship between intelligence and PTSD in adults. While intelligence appears to play a role in the severity and manifestation of PTSD, the exact mechanisms remain unclear. The findings emphasize the need for careful interpretation of cognitive data, consideration of confounding factors, and integration of multiple cognitive domains when assessing trauma outcomes. This review serves as a foundation for future research aimed at clarifying the interplay between intelligence and PTSD, and for developing strategies to support cognitive functioning and recovery in trauma-exposed populations.

This systematic review also has several limitations. First, the review was conducted using three databases: PsycINFO, MEDLINE, and Web of Science. Replicating this review in other databases may yield new insights into the association between intelligence and PTSD.

Second, the sample size for which the studies were conducted is a limitation. The results of this systematic review apply to adults. It would be advisable to conduct a new review focused on the youth or child population to determine whether the conclusions are generalizable across all age groups.

Third, there is a geographical limitation. As mentioned throughout this review, the included studies were exclusively conducted in six countries (the United States, Canada, the Netherlands, Sweden, Denmark and Israel) within three cultural areas (North America, Europe and Jewish Middle East); thus, the results should only be applied to these regions.

Fourth, it is worth mentioning that nine of the studies involved military, ex-military, or police populations, while only three were conducted on civilian populations.

Finally, this review not only synthesizes the information contained in the scientific literature but also serves as a starting point for future research on this topic. Future investigations should prioritize prospective longitudinal designs incorporating pre-trauma assessments of intelligence. Such designs would allow a clearer examination of temporal precedence and help disentangle whether lower intelligence represents a vulnerability factor for PTSD or whether PTSD contributes to subsequent changes in cognitive functioning. Given that most of the existing evidence derives from observational and correlational studies, establishing temporal sequencing is essential to clarify the directionality of the association and to avoid over interpretation of cross-sectional findings.

In addition, greater conceptual and methodological consistency in the assessment of intelligence is warranted. Future studies would benefit from combining standardized measures of general intelligence (e.g., full-scale IQ assessments) with domain-specific neuropsychological evaluations targeting executive functioning, memory, processing speed, and verbal and non-verbal reasoning. This combined approach would facilitate differentiation between global intellectual functioning and specific cognitive domains, thereby improving the interpretability and comparability of results across studies.

Another priority concerns the inclusion of more diverse and representative samples. The current literature is heavily dominated by male military populations from a limited number of Western countries. Future research should expand to civilian populations, include greater female representation, and incorporate culturally diverse samples to determine whether the intelligence–PTSD relationship varies across trauma types, sociocultural contexts, and demographic characteristics. Examining interpersonal, accidental, and community-based trauma in addition to combat-related exposure would further strengthen external validity.

Moreover, future investigations should incorporate more comprehensive control of potential confounding variables, including educational attainment, socioeconomic status, premorbid psychiatric conditions, and comorbid depressive symptomatology. Given the complex and multifactorial nature of PTSD, integrating these variables into analytic models would provide a more nuanced understanding of how cognitive capacity interacts with environmental and psychological risk factors.

Finally, further research integrating cognitive, psychological, and neurobiological perspectives may help clarify the mechanisms underlying this association. Exploring constructs such as cognitive reserve, stress-related neurocognitive changes, and resilience processes may offer deeper insight into how intelligence interacts with trauma exposure and recovery trajectories.

## Figures and Tables

**Table 1:** Results table, which includes the following: author, year of publication, country where the study was developed, study type, sample’s characteristics, tests applied, results, and conclusions.

Author, Year, and Country	Study Type	Participants: Population and Sample	Tests Applied	Results	Conclusions
Burriss et al. (2008) United States	Case–control	American veterans of Vietnam (military sample) *N* = 148 *n*1 (no PTSD + no combat) = 70 Mean age = 56.91 years SD = 14.77 *n*2 (no PTSD + yes combat) = 31 Mean age = 60.59 years *SD* = 13.83 *n*3 (yes PTSD + yes combat) = 47 Mean age = 54.73 years *SD* = 12.83	WAIS: Digit span and vocabulary test WMS-III: Logical Memory (LMMS) and Verbal Paired Associates (VPAS) WAIMI	WAIS (vocabulary): *F*(2, 77) = 4.07/*p* < .02 WAIS (digits): *F*(2, 77) = 0.55/*p* > .1 LMMS: *F*(2, 133) = 5.89/*p* < .01 VPASS: *F*(2, 133) = 5.65/*p* < .001 WAIMI: *F*(2, 133) = 7.91/*p* < .001	WAIS (Vocabulary): Significant differences between *n*1-*n*3 and *n*2-*n*3, but not between *n*1-*n*2. This indicates that the PTSD diagnosis explains the existing differences. WAIS (Digits): No significant differences were observed in any comparison. LMMS: Significant differences between *n*1-*n*2 and *n*1-*n*3, but no differences between *n*2-*n*3. PTSD diagnosis alone does not explain the differences. VPASS: Significant differences between *n*1-*n*3 and *n*2-*n*3, but not between *n*1-*n*2. The PTSD diagnosis explains these differences. WAIMI: Significant differences between *n*1-*n*3 and *n*2-*n*3, but not between *n*1-*n*2. PTSD diagnosis explains these differences. PTSD influences the results of some intelligence scale tests.
Desrochers et al. (2021) Canada	Case–control	Police officers in Quebec’s region (civilian sample) *N* = 61 *n*1 (PTSD) = 31 *n*1.1 (PTSD diagnosis) = 29 *n*1.2 (PTSD subclinical) = 2 *n*2 (control) = 30 Mean age = 40.2 years Years range = 23–63 *SD* = 8.8	TMT: parts A and B WAIS-IV Digit Symbol Test D-KEFS CWIT: conditions 1, 2, 3 and 4 D2 Attention Revision Test RAVLT: first essay. PASAT WMS-III: logical memory types I and II VFT: phoneme “p” and semantics: animals.	Executive functions: *p* = .02/*η*2/*p* = .15 Lexical access: *p* = .001/*η*2/*p* = .19 Verbal learning: *p* = .028/*η*2/*p* = .08 Verbal memory: *p* = .034/*η*2/*p* = .08 Data based on symptom severity: Attention and work memory: *r* = 0.51/*p* < .01 Processing speed: *r* = −0.44/*p* = .03 Executive functions: *r* = 0.42/*p* = .039	Police officers with PTSD had worse outcomes in measurements of Processing Speed: TMT, WAIS-IV, D-KEFS and CWIT. Working Memory and Attention: RAVLT and PASAT. Verbal Learning and Memory: RAVLT, WMS-III. Executive Functions: TMT, VFT, D-KEFS, CWIT. Lexical Access: VFT.
Emdad et al. (2005) Sweden	Case–control	Iraqi refugees in Sweden (civilian sample) *N* = 40 *n*1 (PTSD) = 23 Mean age = 38. 65 years Years range = ± 6.23 *n*2 (control) = 17 Mean age = 37.88 years Years range = ± 8.58	BDT BVRT RSPM TPMT	Correlation between PTSD duration and BDT: *r* = 0.42/*p* = .04, BVRT rotation error: *r* = 0.42/*p* = .04 BVRT distortion error: *r* = 0.41/*p* = .05 Correlation with PTSD severity: RSPM: *r* = 0.42/*p* = .04 TPMT: *r* = 0.44/*p* = .03	Refugees diagnosed with PTSD performed worse on the applied tests, showing a negative correlation in both intelligence scales (RSPM and BDT) and memory scales (BVRT and TPMT) in relation to the duration and severity of the diagnosis.
Gilbertson et al. (2006) United States	Case–control	American Vietnam War veteran (military sample) *N* = 86 *n*1.1 (exposed veterans with PTSD) = 19 *n*1.2 (twins of *n*1.1) = 19 *n*2.1 (veteran exposed without PTSD) = 24 *n*2.2 (twins of *n*2.1) = 24	WAIS-R: Vocabulary, Arithmetic, Picture Completion, and Block Design	IQ measurement: *n*1.1: 104.95/*SD* = 17.33 *n*1.2: 103.47/*SD* = 12.32 *n*2.1: 117.79/*SD* = 16.37 *n*2.2: 117.33/*SD* = 16.37 Effect size for PTSD diagnosis: *d* = 0.92 Effect size for trauma exposure: *d* = 0.14 Correlation between *n*1.1-*n*2.1: *t*(41) = 2.38/*p* = .022 Correlation of PTSD risk among monozygotic twins: not significant.	No statistically significant differences were established between monozygotic twins regarding IQ, but significant differences were found in IQ between exposed veterans with and without a PTSD diagnosis. The main conclusion of the study indicates that IQ may be a risk factor or predisposition.
Macklin et al. (1998) United States	Case–control	American Vietnam War veteran (military sample) *N* = 90 *n*1 (PTSD) = 59 Mean age = 49 years *SD* = 2.4 *n*2 (control) = 31 Mean age = 50 years *SD* = 2.9	AFQT WAIS-R SILS	Veterans with PTSD: Pre-combat intelligence quotient: 106.3/*SD* = 16 Post-diagnosis IQ: 101.4/*SD* = 12 Veterans without PTSD: Pre-combat intelligence quotient: 119/*SD* = 13.3 Post-diagnosis IQ: 109.5/SD = 13.4 Correlation between Pre-combat Intelligence and Combat Exposure: *t*(87) = 6/*p* < .001 Correlation between Pre-combat intelligence and PTSD severity: *r*(88) = −0.45/*p* < .001	Veterans with PTSD had significantly lower pre-combat intelligence compared to the control group. Lower intelligence levels significantly increased combat exposure time. Pre-combat intelligence predicts PTSD severity.
McNally and Robinaugh (2011) United States	Cohort	Adult women exposed to childhood sexual assault (civilian sample) *N* = 100 *n*1 (single abuse by foreigner, non-family or distant relative) = 15 Mean age = 36.1 *n*2 (single abuse by father or brother) = 54 Mean age = 42.4 *n*3 (multiple abuse by father or brother) = 31 Mean age = 45.3	SILS PCL-C	IQ measurement: *n*1 = 98.7/*n*2 = 93.8/*n*3 = 97.6/*F* = 1.037/*p* = .359 Correlation between intelligence and PTSD: *n*1: *r*(13) = −0.68/*p* = .002 *n*2: *r*(46) = 0.15/*p* = .15 *n*3: *r*(47) = −0.001/*p* = .50	Significant differences in intelligence and PTSD were found in women from group *n*1. In contrast, no significant differences were observed in the other groups.
McNally and Shin (1995) United States	Case–control	American Vietnam War veteran (military sample) *N* = 88 *n*1 (PTSD) = 55 *n*2 (control) = 33	WAIS-R	IQ on the WAIS-R Scale: 103.1/*SD* = 14.1 Correlation between IQ and combat exposure: *r* = 0.03/*p* = n.s. Correlation between PTSD Severity and IQ: *r* = 0.32/*df* = 103/*p* = .0005	Vietnam War veterans diagnosed with PTSD showed: 1. Significantly lower scores on the intelligence scale. 2. The same level of exposure to combat situations. 3. The greater the severity of symptoms, the lower the IQ.
Olff et al. (2014) Netherlands	Cohort	Adults residing in the Netherlands (civilian sample) *N* = 56 *n*1 (PTSD) = 28 Mean age = 38.93 years Years range = 19–62 *SD* = 12.25 *n*2 (trauma exposed without PTSD diagnosis) = 28 Mean age = 39.29 years Years range = 23–61 *SD* = 11.48	CANTAB: inhibitory response (SST), cognitive flexibility (IED), working memory (OTS), spatial memory (SWM)	SST measurement: *t*(54) = −2.22/*p* < .05 IED measurement: *t*(33.1) = −3.85/*p* < .05 OTS measurement: Accuracy: *t*(43.4) = −2.39/*p* < .05 Latency: *t*(52) = −3.4/*p* < .05 SWM measurement: Errors: *t*(52) = −3.14/*p* < .05 Strategy efficiency: *t*(46.1) = −3.03/*p* < .05	Participants diagnosed with PTSD obtained significantly lower scores on the CANTAB, including areas of memory that are part of the intelligence measurement.
Sørensen et al. (2016) Denmark	Cohort	Danish soldiers deployed in Afghanistan (military sample) *N* = 743 (initial) *n*1 (T6) = 428 Mean age = 24 *n*1.1 (severe) = 42 *n*1.2 (mid) = 78 *n*1.3 (low) = 308	PCL-C DSM-IV Axis I Børge Priens Prøve (BPP) Traumatic Life Events Questionnaire (TLEQ) Danger/Injury Exposure Score	PCL-C: *n*1 = 428/mean = 24.2/*SD* = 9.1/*n*1.1 (≥44) = 42/*n*1.2(30–43) = 78/*n*1.3 (17–29) = 308 BPP (Cognitive Ability): *n*1 = 428/*n*1.1 = 95 ± 16/*n*1.2 = 102.8 ± 16.4/*n*1.3 = 100.9 ± 14.1/trajectory subset *n*2 = 384/relieved worsening = 86.9 ± 14.5/low stable = 101.4 ± 14.8 Danger/Injury Exposure: *n*1 = 428/mean = 20.9/*SD* = 4.8/range = 10–34; OR (high vs. low quintile) = 3.4 (95% CI 1.43–8.24); unit increase OR = 1.05 (95% CI 0.97–1.14)	Lower pre-deployment cognitive ability is linked to a higher risk of moderate-severe PTSD. Most soldiers exhibit low stable symptom trajectories. Soldiers with lower cognitive scores are more likely to develop severe PTSD symptoms; the relieved-worsening trajectory is characterized by the lowest cognitive ability. Greater perceived war-zone stress increases the likelihood of developing severe PTSD symptoms. Early traumatic life events are considered in analyses, but do not fully explain PTSD risk.
Vasterling et al. (1997) United States	Case–control	Gulf War veterans (military sample) *N* = 41 *n*1 (PTSD) = 18 Mean age = 35.17 *SD* = 9.8 *n*2 (control) = 23 Mean age = 34.8 years *SD* = 8.4	WAIS-R: verbal (VIQ) and performance (PIQ)	*n*1: FIQ = 90.1/VIQ = 91.1/PIQ = 90.9 *n*2: FIQ = 97.9/VIQ = 98.7/PIQ = 97.7 FSIQ: *r* = 0.37/*p* = .022 VIQ: *F*(6, 34) = 4.31/*p* = .002/*r* = −0.39/*p* = .016 PIQ: *F*(5, 35) = 1.58/*p* = .19/*r* = 0.3/*p* > .06	Veterans diagnosed with PTSD scored lower in general intelligence and in the verbal and performance areas. However, the differences in performance were not significant.
Vasterling et al. (2002) United States	Case–control	American Vietnam War veteran (military sample) *N* = 47 *n*1 (PTSD) = 26 Mean age = 50.19 *SD* = 3.28 *n*2 (control) = 21 Mean age = 51.57 years *SD* = 4.91	WAIS-R: Information and vocabulary assessment	*n*1: WAIS-information: 10.5/*SD* = 2.4 WAIS vocabulary: 10.3/*SD* = 2.4 *n*2: WAIS information: 1.2/*SD* = 2.4 WAIS vocabulary: 11.8/*SD* = 2.4 Differences comparing the results obtained with the EPIQ (estimated IQ before exposure): *F*(1, 45) = 5.84/*p* < .02	Significant differences were found in terms of the difference between the current intelligence measurement and EPIQ based on the PTSD diagnosis.
Zalmenson et al. (2024) Israel	Cohort	Male Israeli soldiers (military sample) *N* = 582 Mean age = 19.6 *SD* = 0.8 Range = 18–27 *n*1 (15 months) = 484 *n*2 (27 months) = 400	IDF (General IQ pre-military assessment) Combat Exposure Scale (repeated measures) PCL-5 Non-verbal abstract reasoning subscale PTSD symptoms GAD-7 PHQ	IDF (General IQ pre-military assessment) TIME × IQ = −0.05/*SE* = 0.02/*t* (442.79) = −3.26/*p* < .01 TIME × IQ (Combat Exposure) = −0.02/*SE* = 0.004/*t* (520.84) = −4.52/*p* < .0001 CES (Combat Exposure Scale) *β* = 0.41/*p* < .001 PCL-5 (PTSD symptoms) *p* < .01 GAD-7 (anxiety) *β* = −0.28/*p* < .01 PHQ (depression) *r* = −0.25/*p* < .01	Lower IQ predicted steeper PTSD symptom growth ×3.5 times and higher risk; higher IQ showed a protective effect ×12.4 times. Combat exposure mediated part of the relationship between IQ and PTSD. PTSD symptoms increased more steeply among lower-IQ participants. Lower IQ is associated with higher anxiety and depression levels.

Note: The abbreviations used in this table correspond to the following psychometric tests. Armed Forces Qualification Test (AFQT); Benton Visual Retention Test (BVRT); Block Design Test (BDT); Børge Priens Prøve (BPP); Cambridge Neuropsychological Test Automated Battery (CANTAB); Colour–Word Interference Test (CWIT); Combat Exposure Scale (CES); Danger and Injury Exposure Scale (DIES); Delis–Kaplan Executive Function System (D-KEFS); Diagnostic and Statistical Manual of Mental Disorders, Fourth Edition (DSM-IV); Generalized Anxiety Disorder 7-item scale (GAD-7); Paced Auditory Serial Addition Test (PASAT); Patient Health Questionnaire (PHQ); Posttraumatic Checklist civilian version (PCL-C); Raven’s Standard Progressive Matrices (RSPM); Rey Auditory Verbal Learning Test (RAVLT); Shipley Institute of Living Scale (SILS); Traumatic Life Events Questionnaire (TLEQ); Thurstone’s Picture Memory Test (TPMT); Trail Making Test (TMT); Verbal Fluency Task (VFT); Wechsler Adult Intelligence Scale (WAIS); Wechsler Adult Intelligence Scale (WAIS-R); Wechsler Adult Intelligence Scale Fourth Edition (WAIS-IV); Wechsler Auditory Immediate Memory Index (WAIMI); Wechsler Memory Scale (WMS-III).

**Table 2:** Bias Risk Assessment (Cochrane Collaboration; Higgins & Green, 2011).

	Selection Bias		Performance Bias	Detection Bias	Attrition Bias	Reporting Bias	Other Biases
	Random Sequence Generation	Allocation Concealment	Blinding of Participants and Personnel	Blinding of Outcome Assessors	Handling of Incomplete Outcome Data	Selective Reporting	Limitations
Burriss et al. (2008)	Low risk	Not specified	Not specified	Not specified	Low risk	Low risk	Low risk
Desrochers et al. (2021)	Low risk	Not specified	Not specified	Not specified	Low risk	Low risk	Low risk
Emdad et al. (2005)	High risk	Not specified	Not specified	Low risk	Low risk	Low risk	High risk
Gilbertson et al. (2006)	High risk	Not specified	Low risk	Not specified	Low risk	Low risk	Low risk
Macklin et al. (1998)	High risk	Not specified	Not specified	Not specified	Low risk	Low risk	Low risk
McNally and Robinaugh (2011)	Low risk	Not specified	Not specified	Not specified	Low risk	Low risk	Low risk
McNally and Shin (1995)	Not specified	Not specified	Not specified	Not specified	Low risk	Not specified	Low risk
Olff et al. (2014)	Low risk	Not specified	Not specified	Not specified	Low risk	Low risk	Low risk
Sørensen et al. (2016)	High risk	Not specified	Not specified	Not specified	Low risk	Low risk	Low risk
Vasterling et al. (1997)	High risk	Not specified	Not specified	Not specified	Low risk	Low risk	Low risk
Vasterling et al. (2002)	High risk	Not specified	Not specified	Not specified	Low risk	Low risk	Low risk
Zalmenson et al. (2024)	High risk	Not specified	Not specified	Not specified	Low risk	Low risk	Low risk

## Data Availability

No datasets were generated or analyzed during the current study.

## Supplementary Materials

The following supporting information can be downloaded at https://www.mdpi.com/article/10.3390/jintelligence14050073/s1, Table S1: PRISMA 2020 Ckecklist.

## Abbreviations

The following abbreviations are used in this manuscript:

AFQT	Armed Forces Qualification Test
BDT	Block Design Test
BPP	Børge Priens Prøve
BVRT	Benton Visual Retention Test
CANTAB	Cambridge Neuropsychological Test Automated Battery
CES	Combat Exposure Scale
CWIT	Colour–Word Interference Test
D-KEFS	Delis–Kaplan Executive Function System
DIES	Danger and Injury Exposure Scale
DSM-IV	Diagnostic and Statistical Manual of Mental Disorders, Fourth Edition
GAD-7	Generalized Anxiety Disorder 7-item scale
IQ	Intelligence quotient
PASAT	Paced Auditory Serial Addition Test
PCL-C	Posttraumatic Checklist—Civilian Version
PHQ	Patient Health Questionnaire
PROSPERO	International Prospective Register of Systematic Reviews
PTSD	Posttraumatic stress disorder
RAVLT	Rey Auditory Verbal Learning Test
RSPM	Raven’s Standard Progressive Matrices
SILS	Shipley Institute of Living Scale
TLEQ	Traumatic Life Events Questionnaire
TMT	Trail Making Test
TPMT	Thurstone’s Picture Memory Test
VFT	Verbal Fluency Task
WAIMI	Wechsler Auditory Immediate Memory Index
WAIS	Wechsler Adult Intelligence Scale
WAIS-IV	Wechsler Adult Intelligence Scale, Fourth Edition
WAIS-R	Wechsler Adult Intelligence Scale—Revised
WMS-III	Wechsler Memory Scale, Third Edition

## References

[B1-jintelligence-14-00073] Al Jowf G. I., Ahmed Z. T., Reijnders R. A., de Nijs L., Eijssen L. M. T. (2025). To predict, prevent, and manage post-traumatic stress disorder (PTSD): A review of pathophysiology, treatment, and biomarkers. International Journal of Molecular Sciences.

[B2-jintelligence-14-00073] American Psychiatric Association (2013). Diagnostic and statistical manual of mental disorders.

[B3-jintelligence-14-00073] Badenes-Ribera L., Molla-Esparza C., Longobardi C., Sánchez-Meca J., Fabris M. A. (2021). Homicide as a source of posttraumatic stress? A meta-analysis of the prevalence of posttraumatic stress disorder after committing homicide. Journal of Traumatic Stress.

[B4-jintelligence-14-00073] Bonanno G. A., Mancini A. D. (2012). Beyond resilience and PTSD: Mapping the heterogeneity of response to potential trauma. Psychological Trauma: Theory, Research, Practice, and Policy.

[B5-jintelligence-14-00073] Brewin C. R., Andrews B., Valentine J. D. (2000). Meta-analysis of risk factors for posttraumatic stress disorder in trauma-exposed adults. Journal of Consulting and Clinical Psychology.

[B6-jintelligence-14-00073] Burriss L., Ayers E., Ginsberg J., Powell D. A. (2008). Learning and memory impairment in PTSD: Relationship to depression. Depression and Anxiety.

[B7-jintelligence-14-00073] Charney D. S. (2004). Psychobiological mechanisms of resilience and vulnerability: Implications for successful adaptation to extreme stress. The American Journal of Psychiatry.

[B8-jintelligence-14-00073] Colom R., Ma P. C. S. (2024). Cognitive ability, personality, and psychopathology: A stormy relationship. Journal of Intelligence.

[B9-jintelligence-14-00073] Deary I. J. (2012). Intelligence. Annual Review of Psychology.

[B10-jintelligence-14-00073] Desrochers A. B., Rouleau I., Angehern A., Vasiliadis H. M., Saumier D., Brunet A. (2021). Trauma on duty: Cognitive functioning among police officers with and without posttraumatic stress disorder (PTSD). European Journal of Psychotraumatology.

[B11-jintelligence-14-00073] Durao M., Etchezahar E., Albalá Genol M. Á., Muller M. (2023). Fear of missing out, emotional intelligence and attachment in older adults in Argentina. Journal of Intelligence.

[B12-jintelligence-14-00073] Emdad R., Sondergaard H. P., Theorell T. (2005). Learning problems, impaired short-term memory, and general intelligence related to disease severity and duration in posttraumatic stress disorder patients. Stress, Trauma, and Crisis.

[B13-jintelligence-14-00073] Gardner H. (1987). Estructuras de la mente: La teoría de las múltiples inteligencias *[Frames of mind: The theory of multiple intelligences]*.

[B14-jintelligence-14-00073] Gervasio M., Beatty A., Kavanaugh B., Cancilliere M. K., Holler K. (2022). The association between neurocognition and sexual abuse within a children’s psychiatric inpatient program. The Clinical Neuropsychologist.

[B15-jintelligence-14-00073] Gilbertson M. W., Paulus L. A., Williston S. K., Gurvits T. V., Lasko N. B., Pitman R. K., Orr S. P. (2006). Neurocognitive function in monozygotic twins discordant for combat exposure: Relationship to posttraumatic stress disorder. Journal of Abnormal Psychology.

[B16-jintelligence-14-00073] Hahnefeld A., Sukale T., Weigand E., Dudek V., Münch K., Aberl S., Eckler L. V., Nehring I., Friedmann A., Plener P. L., Fegert J. M., Mall V. (2022). Non-verbal cognitive development, learning, and symptoms of PTSD in 3- to 6-year-old refugee children. European Journal of Pediatrics.

[B17-jintelligence-14-00073] Higgins J. P. T., Green S. (2011). Cochrane handbook for systematic reviews of interventions *(Version 5.1.0)*.

[B18-jintelligence-14-00073] Kremen W. S., Koenen K. C., Boake C., Purcell S., Eisen S. A., Franz C. E., Tsuang M. T., Lyons M. J. (2007). Pretrauma cognitive ability and risk for posttraumatic stress disorder: A twin study. Archives of General Psychiatry.

[B19-jintelligence-14-00073] Macklin M. L., Metzger L. J., Litz B. T., McNally R. J., Lasko N. B., Orr S. P., Pitman R. K. (1998). Lower precombat intelligence is a risk factor for posttraumatic stress disorder. Journal of Consulting and Clinical Psychology.

[B20-jintelligence-14-00073] McEwen B. S. (2007). Physiology and neurobiology of stress and adaptation: Central role of the brain. Physiological Reviews.

[B21-jintelligence-14-00073] McNally R. J., Robinaugh D. J. (2011). Risk factors and posttraumatic stress disorder: Are they especially predictive following exposure to less severe stressors?. Depressive and Anxiety.

[B22-jintelligence-14-00073] McNally R. J., Shin L. M. (1995). Association between intelligence and the severity of posttraumatic stress disorder symptoms among Vietnam combat veterans. American Journal of Psychiatry.

[B23-jintelligence-14-00073] Neisser U., Boodoo G., Bouchard T. J., Boykin A. W., Brody N., Ceci S. J., Halpern D. F., Loehlin J. C., Perloff R., Sternberg R. J., Urbina S. (1996). Intelligence: Knowns and unknowns. American Psychologist.

[B24-jintelligence-14-00073] Ogata K. (2017). Maltreatment related trauma symptoms affect academic achievement through cognitive functioning: A preliminary examination in Japan. Journal of Intelligence.

[B25-jintelligence-14-00073] Olff M., Polak A. R., Witteveen A. B., Denys D. (2014). Executive function in posttraumatic stress disorder (PTSD) and the influence of comorbid depression. Neurobiology of Learning and Memory.

[B26-jintelligence-14-00073] Ozer E. J., Best S. R., Lipsey T. L., Weiss D. S. (2003). Predictors of posttraumatic stress disorder and symptoms in adults: A meta-analysis. Psychological Bulletin.

[B27-jintelligence-14-00073] Page M. J., McKenzie J. E., Bossuyt P. M., Boutron I., Hoffmann T. C., Mulrow C. D., Shamseer L., Tetzlaff J. M., Akl E. A., Brennan S. E., Chou R., Glanville J., Grimshaw J. M., Hróbjartsson A., Lalu M. M., Li T., Loder E. W., Mayo-Wilson E., McDonald S., Moher D. (2021). The PRISMA 2020 statement: An updated guideline for reporting systematic reviews. BMJ.

[B28-jintelligence-14-00073] Rock P. L., Roiser J. P., Riedel W. J., Blackwell A. D. (2014). Cognitive impairment in depression: A systematic review and meta-analysis. Psychological Medicine.

[B29-jintelligence-14-00073] Silverio S. A., Lyons M. T., Burton S. P. (2023). Dangerously intelligent: A call for re-evaluating psychopathy using perceptions of intelligence. Journal of Intelligence.

[B30-jintelligence-14-00073] Sørensen H. J., Andersen S. B., Karstoft K. I., Madsen T. (2016). The influence of pre-deployment cognitive ability on post-traumatic stress disorder symptoms and trajectories: The Danish USPER follow-up study of Afghanistan veterans. Journal of Affective Disorders.

[B31-jintelligence-14-00073] Spearman C. (1904). “General intelligence,” objectively determined and measured. The American Journal of Psychology.

[B32-jintelligence-14-00073] Sternberg R. J. (1985). Beyond IQ: A triarchic theory of intelligence.

[B33-jintelligence-14-00073] Tedeschi C. G., Calhoun L. G. (2004). Posttraumatic growth: Conceptual foundations and empirical evidence. Psychological Inquiry.

[B34-jintelligence-14-00073] Tolin D. F., Foa E. B. (2006). Sex differences in trauma and PTSD: A quantitative review. Psychological Bulletin.

[B35-jintelligence-14-00073] Vasterling J. J., Brailey K., Constans J. I., Borges A., Sulke P. B. (1997). Assessment of intellectual resources in gulf war veterans: Relationship to PTSD. Assessment.

[B36-jintelligence-14-00073] Vasterling J. J., Duke L. M., Brailey K., Constans J. I., Allain A. N., Sutker P. B. (2002). Attention, learning, and memory performances and intellectual resources in Vietnam veterans: PTSD and no disorder comparisons. Neuropsychology.

[B37-jintelligence-14-00073] Wechsler D. (1958). The measurement and appraisal of adult intelligence.

[B38-jintelligence-14-00073] Wechsler D. (2008). WAIS-IV: Wechsler adult intelligence scale—Fourth edition: Technical and interpretive manual.

[B39-jintelligence-14-00073] Williams C. M., Peyre H., Labouret G., Fassaya J., Guzmán García A., Gauvrit N., Ramus F. (2023). High intelligence is not associated with a greater propensity for mental health disorders. European Psychiatry.

[B40-jintelligence-14-00073] Yehuda R. (2002). Post-traumatic stress disorder. New England Journal of Medicine.

[B41-jintelligence-14-00073] Zalmenson T., Yair N., Azriel O., Shamai-Leshem D., Alon Y., Tik N., Levinstein Y., Ben-Yehuda A., Tatsa-Laur L., Pine D. S., Bliese P. D., Tavor I., Bar-Haim Y. (2024). The effects of intelligence on exposure to combat and posttraumatic stress disorder across multiple deployments. Journal of Anxiety Disorders.

